# Transglycosylation Activity of Glycosynthase Mutants of Endo-β-*N*-Acetylglucosaminidase from *Coprinopsis cinerea*

**DOI:** 10.1371/journal.pone.0132859

**Published:** 2015-07-21

**Authors:** Yasunari Eshima, Yujiro Higuchi, Takashi Kinoshita, Shin-ichi Nakakita, Kaoru Takegawa

**Affiliations:** 1 Department of Bioscience and Biotechnology, Faculty of Agriculture, Kyushu University, 6-10-1 Hakozaki, Fukuoka, Japan; 2 Fushimi Pharmaceutical Co. Ltd., Marugame, Kagawa, Japan; 3 Department of Functional Glycomics, Life Science Research Center, Kagawa University, Miki-cho, Kagawa, Japan; University of Insubria, ITALY

## Abstract

Endo-β-*N*-acetylglucosaminidase (ENGase), which catalyzes hydrolysis of *N*-linked oligosaccharides, is a useful tool for analyzing oligosaccharide contents of glycoproteins. However, there are only a few known ENGases that can catalyze the hydrolysis of human complex type oligosaccharides, and although commercially available, they are expensive. Here, we report the cloning of two ENGase encoding cDNAs from the basidiomycete fungus *Coprinopsis cinerea*, Endo-CC1 and Endo-CC2. We successfully expressed recombinant His_6_-tagged Endo-CC1 and Endo-CC2 in *Escherichia coli* and purified them for enzymatic characterization. Both Endo-CC1 and Endo-CC2 showed hydrolytic activity on high-mannose and complex type oligosaccharides. Since Endo-CC1 could be prepared more easily than Endo-CC2 from *E*. *coli* cultures, we examined the enzymatic properties of Endo-CC1 in detail. Our results showed that Endo-CC1 acted on both *N*-linked high-mannose type and sialobiantennary type complex oligosaccharides of glycoproteins RNase B and human transferrin, respectively, but not on the sialotriantennary type complex oligosaccharide of glycoprotein fetuin. Examination of the transglycosylation activity of Endo-CC1 revealed that the wild-type Endo-CC1 could not transfer the sialobiantennary type complex oligosaccharide onto the deglycosylated RNase B. To obtain an Endo-CC1 mutant with desired transglycosylation activity, we performed mutation analysis of the active site residue Asn 180 (N180), known to be important for catalysis, by individually replacing it with the remaining 19 amino acid residues. Transglycosylation analyses of these mutants led us to identify one mutant, namely Endo-CC1^N180H^, which exhibited the desired transglycosylation activity. Taken together, we suggest that Endo-CC1 would potentially be a valuable tool for analyzing oligosaccharides on glycoproteins, as large quantities of it could be made available more easily and less expensively than the currently used enzyme, Endo-M.

## Introduction

In eukaryotic cells, most of the secreted proteins are generally modified with sugar chains, which are crucial for their stability and bioactivity [1,2]. Specifically, *N*-linked glycans are found to be attached to glycoproteins via the nitrogen atom of the side chain of aspargine residue in the specific sequence N-X-S/T (X, any amino acid except P). Although many glycoproteins are potential biopharmaceuticals, bioactivities of these glycoproteins are not consistent because of the heterogeneous natures of their *N*-linked oligosaccharides [3,4]. Thus, to make the bioactivities of pharmaceutically important glycoproteins consistent, it is necessary that their *N*-linked glycan structures are homogenous. One of the methods for achieving this goal is to attach homogeneous oligosaccharides onto glycoproteins, original heterogeneous *N*-linked glycans of which are already removed by enzymatic treatment [5–8]. In this chemoenzymatic strategy, enzymes that could effectively catalyze *N*-linked oligosaccharides are highly sought after.

Endo-β-*N*-acetylglucosaminidase (ENGase) hydrolyzes the *N*, *N’*-diacetylchitobiose moiety of the *N*-linked oligosaccharides on glycoproteins. ENGases are divided into two groups: glycosyl hydrolase family 18 (GH18) and glycosyl hydrolase family 85 (GH85). In addition to the hydrolase activity, the GH85 ENGases, such as Endo-A from *Arthrobacter protophormiae* [9–11] and Endo-M from *Mucor hiemalis* [12–14], but not the GH18 ENGases, have been shown to exhibit transglycosylation activity. Whereas Endo-A can use the high-mannose type, but not the complex type, oligosaccharide as a donor substrate, Endo-M can use both types as donor substrates for transglycosylation. This transglycosylation activity of Endo-M is useful for producing pharmaceutical glycoproteins, in which the attached oligosaccharides are homogeneously modified, a requirement critical for their activity. However, commercially available Endo-M is a relatively expensive enzyme, as high amount of it could not be prepared from the culture of *E*. *coli* expressing recombinant Endo-M [15], thereby limiting its application.

To circumvent this problem, we searched for novel ENGases that can be prepared easily and in abundant quantities from *E*. *coli* culture. We identified two candidate ENGases in *Coprinopsis cinerea*, namely Endo-CC1 and Endo-CC2, and characterized their enzymatic properties in detail. Both enzymes catalyzed the hydrolysis of high-mannose and complex type oligosaccharides. Furthermore, based on the mutational analysis of Endo-CC1, we have created an Endo-CC1 mutant, N180H, that could use sialobiantennary complex type oligosaccharide as a donor substrate for transglycosylation.

## Materials and Methods

### Strains and media

*E*. *coli* strain BL21-CodonPlus (DE3) (Stratagene) and the expression vector pET23b (Novagen, USA) were used for all recombinant DNA cloning experiments in this study. For pre- and main cultures, *E*. *coli* cells were grown at 37°C and 30°C in media MMIAC (1.25% triptone, 2.5% yeast extract, 0.85% NaCl, 0.4% glycerol, 20 mM Tris-HCl pH 7.2, 30 mg/L ampicillin and chloramphenicol) and LBAC (2.5% LB powder (Merck), 30 mg/L ampicillin and chloramphenicol), respectively. *Coprinopsis cinerea* strain Okayama-7 (FGSC 9003) was purchased from ATCC.

### Cloning of cDNAs coding for Endo-CCs from *Coprinopsis cinerea*

*C*. *cinerea* strain Okayama-7 was cultured on matsutake agar plates (0.5% EBIOS tablet (Asahi beer), 2% glucose, 2% agar) at 20°C for 2 weeks. Five 1 x 1 cm squares of mycelia were harvested from the matsutake agar plates and further cultured in 100 ml matsutake liquid medium at 100 rpm for 1 week at 20°C. Cells were collected by centrifugation, collected cells were frozen at -80°C for 1 h, frozen cells were broken using the metal cone of a Multi-Beads Shocker (Yasui Kikai) and subsequently they were used for the isolation of total RNA, which was achieved with RNAiso (Takara). cDNAs were prepared from the total RNA using ReverseTra Ace qPCR RT Master Mix (Toyobo). An aliquot of this cDNA mixture was used for amplifying the Endo-CC1 and Endo-CC2 encoding cDNAs by PCR using PrimeSTAR HS DNA polymerase (Takara) and two PCR primers; one of the primers harbored an Nde I restriction enzyme site whereas the other harbored a Xho I restriction enzyme site to facilitate the cloning of the amplified fragment into the Nde I/Xho I digested pET23b vector. For mutagenesis of Endo-CC1, we amplified point-mutated sequences of Endo-CC1 by inverse PCR using PrimeSTAR MAX DNA polymerase (Takara), primers containing each point mutation and the pET23b vector harboring the native Endo-CC1 sequence as a template. Each amplicon was self-ligated using In-Fusion HD Cloning Kit (Takara). Each induced mutation of all resultant plasmids was confirmed by the DNA sequencing. Primers used to clone the native Endo-CCs and primers used for creating the point mutants of Endo-CC1 are summarized in S1 Table.

### Sequence analysis

Amino acid sequences of ENGases were retrieved from the NCBI database. Accession numbers of these ENGases are as follows: Endo-CC1, XP_001839402; Endo-CC2, XP_002911817; Endo-A, AAD10851.1; Endo-BH, WP_010896958.1; Endo-D, BAB62042.1; Endo-M, BAB43869.1; Endo-F1, WP_034866176.1; Endo-F2, WP_034868772.1; Endo-F3, WP_034868774.1; Endo-FV, ACV60538.1; Endo-H, P04067.1; and Endo-S, WP_011285695.1. A phylogenic tree was generated by the neighbor-joining method using the MEGA 6.06 program [16].

### Purification of recombinant Endo-CCs

*E*. *coli* cells expressing His_6_-tagged Endo-CC1 or Endo-CC2 were precultured in MMIAC liquid medium at 37°C for 12 h, the preculture was inoculated into 250 ml of LBAC liquid medium, OD_600_ of cells was adjusted to 0.01 and cells were cultured at 30°C for another 12 h. After collecting the cells by centrifugation at 7000 x *g* for 7 min at 4°C, the pellet was suspended in 5 ml of breaking buffer (300 mM NaCl, 200 mM Tris-HCl pH 7.5). Resuspended cells were lysed by ultrasonication on ice and then centrifuged at 15400 x *g* for 10 min at 4°C. His_6_-tagged Endo-CCs were purified from the supernatant using a HisTrapTM FF 1 ml column (GE Healthcare) and 20 mM Tris-HCl pH 7.5 buffer according to the manufacture’s instructions. Resultant protein samples were concentrated by ultrafiltration using Amicon Ultra 0.5 ml filters (Millipore). Protein concentration was measured using the BCA Protein Assay Kit (Takara). The concentrated protein was subsequently used in the activity analysis.

### Analysis of hydrolase activity of Endo-CCs

The hydrolase activity of Endo-CCs was analyzed by TLC. Briefly, 30 ng of either recombinant Endo-CC1 or recombinant Endo-CC2 in 100 mM phosphate buffer (pH 7.5) was mixed with 1 mM of either dansyl chloride (Dns)-labeled Man_5_GlcNAc_2_-Asn or Neu_2_Gal_2_GlcNAc_2_Man_3_GlcNAc_2_-Asn (Dns-SG; Fushimi Pharmaceutical Co.) in a total volume of 10 μL and the mixture was incubated overnight at 37°C. The reaction samples were spotted onto a 10 cm long TLC Silica gel 60 plate (Merck) and separated using 1-butanol/acetic acid/water (2:2:1, v/v) as the solvent. The plate was dried and the hydrolyzed Dns-Asn-GlcNAc on the plate was detected using LAS-4000 mini (Fujifilm).

To determine the substrate specificities of Endo-CC1 and Endo-CC2, 30 ng of the enzyme was mixed with 2 pmol of each pyridylamino (PA)-labeled-oligosaccharide (Masuda Chemical) in 10 μL of 100 mM phosphate buffer (pH 7.5) and the mixture was incubated at 37°C for 10 min, following which the reaction was stopped by the addition of 2 μl 10% trichloro acetic acid (TCA). The resultant samples were analyzed by HPLC (GL Science), which was equipped with a Wakosil 5C18 column (Wako), set at 40°C. The HPLC was conducted using 50 mM ammonium acetate buffer (pH 4.0) and 0.15% 1-butanol (Nacalai tesque) at the flow rate of 1.5 mL/min. Fluorescence emitted from PA (excitation at 320 nm, emission at 400 nm) was monitored, and the relative hydrolytic activity of Endo-CC1 or Endo-CC2 for each substrate was determined from the peak area of hydrolyzed PA-GlcNAc.

The optimum pH for the hydrolase activity of Endo-CC1 was assessed using Dns-SG as a substrate and 100 mM acetate buffer (for varying the pH from 4.0 to 6.0 in increments of 0.5 pH) or 100 mM phosphate buffer (for varying the pH from 6.0 to 8.0 in increments of 0.5 pH). The reaction was performed as follows: 0.2 mM of Dns-SG was mixed with 30 μg Endo-CC1 in 10 μL of a 200 mM buffer (at an indicated pH) and incubated at 37°C for 3 min, following which the reaction was terminated with 2 μl of 10% TCA. The resultant reaction samples were analyzed by HPLC, which was equipped with a Wakosil 5C18 column, set at 30°C. The HPLC was done using a mixture of 25 mM borate buffer (pH 7.5) and 17% acetonitrile at the flow rate of 1 mL/min. Fluorescence emitted from Dns (excitation at 313 nm, emission at 540 nm) was analyzed, and the relative hydrolase activity was determined from the peak area of hydrolyzed Dns-Asn-GlcNAc. Relative activities of all Endo-CC1 mutants were determined in the manner for measuring the optimum pH. To determine the Km for Dns-SG, the relative activity of Endo-CC1 was analyzed as described above at various Dns-SG concentrations (0.125, 0.143, 0.167, 0.25, 0.33, 0.5, 1.0 or 2.0 mM) in 10 μL of 100 mM phosphate buffer (pH 7.5). Based on known molar extinction coefficient value of Dns-Asn-GlcNAc at 213 nm (3.7 x 10^4^ mol/L [17]), a standard curve was prepared and the Km value was calculated from the Lineweaver-Burk plot of the experimental data points. To determine the thermostability of Endo-CC1, a solution containing 3 ng/μl of Endo-CC1 was incubated for 10 min at various temperatures (35°C-60°C at 5°C intervals) and the relative activity was measured as described above.

To investigate whether Endo-CC1 could hydrolyze glycoproteins, 5 μg of recombinant Endo-CC1 in 50 μL of 100 mM phosphate buffer (pH 6.0) was incubated overnight with either 10 μg of RNase B (Sigma), human transferrin (Sigma) or fetuin (Sigma) at 37°C. The reaction samples were analyzed by SDS-PAGE and the gels were stained with Coomassie Brilliant Blue (CBB) EzStain AQua (Atto).

### Assay for transglycosylation activity of Endo-CC1

To determine the transglycosylation activity of native or point-mutated Endo-CC1, deglycosylated-RNase B (GlcNAc-RNase B) was first prepared as follows: 20 μg of recombinant Endo-CC1 was incubated overnight with 2 mg of RNase B in 1 mL of 25 mM phosphate buffer (pH 7.5) at 37°C, and thereafter the reaction mixture was concentrated by ultrafiltration using an Amicon Ultra filter to obtain a 20 μg/μL solution of GlcNAc-RNase B. For the transglycosylation reaction, the prepared GlcNAc-RNase B and sialyl glycopeptide (SGP, H-Lys-Val-Ala-Asn[(NeuAc-Gal-GlcNAc-Man)_2_-Man-GlcNAc_2_]-Lys-Thr-OH; Fushimi Pharmaceutical) were used as acceptor and donor substrates, respectively. The reaction was carried out using 3 μg of Endo-CC1 (wild-type or mutant), 40 μg of GlcNAc-RNase B and indicated amounts of SGP in 25 mM phosphate buffer (pH 7.5), in 10 μL total volume, at 30°C for 1 or 12 h. The resultant samples were analyzed by SDS-PAGE and the gels were subsequently stained with CBB EzStain AQua to check whether Neo-RNase B was generated.

### Analysis of Neo-RNase B

First, to obtain sufficient amount of Neo-RNase B, 400 μg of GlcNAc-RNase B, prepared as described above, was mixed with 15 μg of Endo-CC1^N180H^ and 1 mg of SGP in 25 mM phosphate buffer (pH 7.5), in a total volume of 100 μL, and incubated at 30°C for 12 h. Subsequently, the unreacted GlcNAc-RNase B was removed with a HiTrap Con A 4B column (GE Healthcare), and the volume of the resultant purified Neo-RNase B sample was concentrated to 40 μL by ultrafiltration. Thereafter, 10 μL of the purified Neo-RNase B solution in 25 mM phosphate buffer (pH 7.5) was mixed with 1 μg of either Endo-A (provided by Dr. Fujita in Kagoshima University) or Endo-CC1, in a total volume of 20 μL, and incubated at 30°C for 1 h. The reaction samples were analyzed by SDS-PAGE and subsequently the gels were stained with CBB EzStain AQua.

## Results

### Cloning of Endo-CC encoding cDNAs

**Fig 1:**
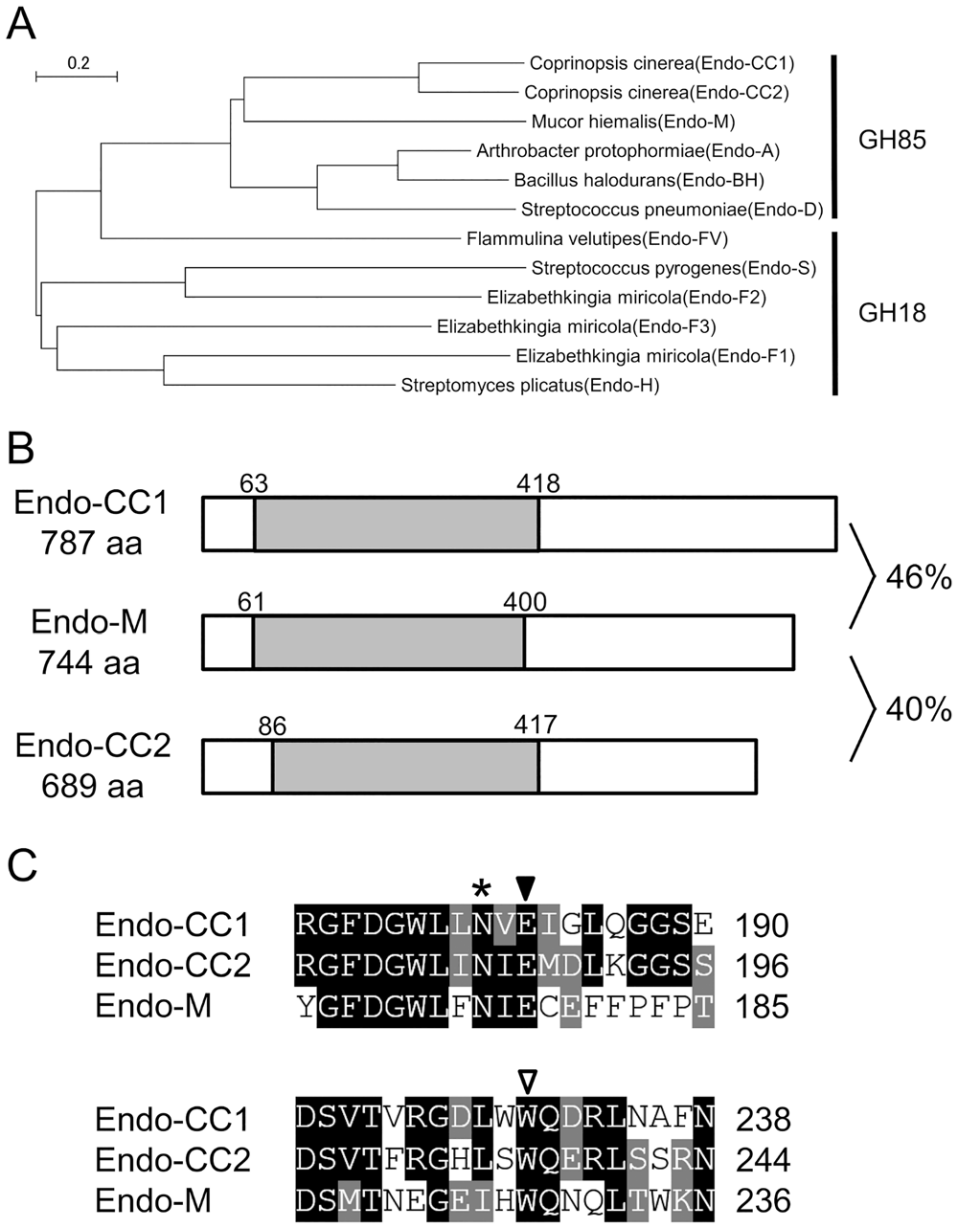
Sequence alignment of Endo-CCs and Endo-M. (A) Phylogenetic tree of ENGases belonging either to GH85 or to GH18 family was generated by the neighbor-joining method and using the MEGA 6.02 program [16]. Endo-CC1 and Endo-CC2 are assigned in the same clade with Endo-M. Note that Endo-CC1, Endo-CC2 and Endo-M are from fungi, whereas Endo-A, Endo-BH and Endo-D are from bacteria. Accession numbers of proteins used in this study are given in Materials and Methods. (B) Endo-CC1 and Endo-CC2 are consisted of 787 and 689 amino acid residues, and show 46% and 40% similarities to Endo-M, respectively. GH85 domain (gray box) is conserved among these proteins. (C) Alignment of amino acid sequences of Endo-CC1, Endo-CC2 and Endo-M around the predicted active site is shown. Glutamic acid (indicated with a closed triangle) and tryptophan (indicated with an open triangle) residues are known to be important for the catalysis and transglycosylation activities, respectively. The asparagine residue, indicated with an asterisk, was subjected to point mutation analyses.

To search for a novel ENGase that can catalyze the hydrolysis and transglycosylation of complex type oligosaccharides, we conducted *in silico* analysis of amino acid sequences of ENGases from several organisms. As a result of this analysis, we discovered two putative ENGases in the basidiomycete *C*. *cinerea* and named them Endo-CC1 and Endo-CC2 (Fig 1A). We next cloned the cDNAs coding for the Endo-CC1 and Endo-CC2 from *C*. *cinerea* cells, and confirmed that Endo-CC1 (consisting of 787 amino acid residues) and Endo-CC2 (consisting of 689 amino acid residues) exhibit 46% and 40% similarities to Endo-M, respectively (Fig 1B). Both of them contain the amino acid sequence of glycosyl hydrolase family 85, according to Pfam 27.0 program (http://pfam.xfam.org/). Our sequence alignment analysis also revealed that glutamic acid and tryptophan residues, which are known to be crucial for catalysis and transglycosylation of Endo-M, respectively [18], are conserved in Endo-CC1 and Endo-CC2 (Fig 1C), suggesting that both Endo-CC1 and Endo-CC2 would most likely exhibit Endo-M-like ENGase activity.

### Characterization of hydrolase activity of Endo-CCs

**Fig 2:**
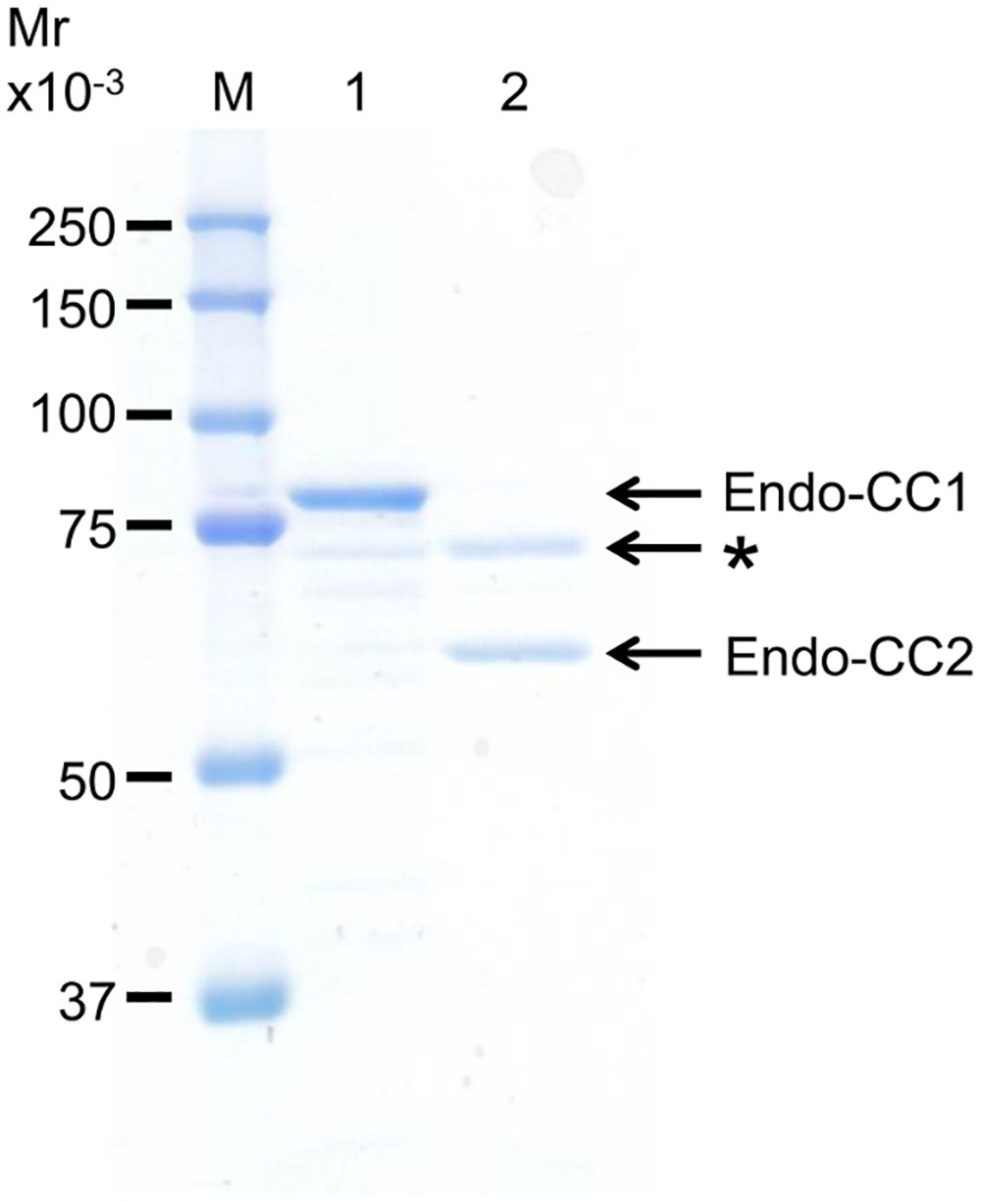
SDS-PAGE analysis of purified Endo-CCs. Endo-CC1 and Endo-CC2, expressed in *E*. *coli*, were purified and then 1 μg of these protein samples were loaded onto a 10% acrylamide gel. The band marked with an asterisk in lane 2 is likely a contaminating *E*. *coli* protein. Lane M, molecular weight markers; lane 1, purified recombinant Endo-CC1; lane 2, purified recombinant Endo-CC2.

To characterize the hydrolase activity of Endo-CCs, we first individually expressed Endo-CC1 and Endo-CC2 cDNAs in *E*. *coli*, and successfully purified recombinant Endo-CC1 and Endo-CC2 proteins from the respective *E*. *coli* cultures grown at 30°C, as judged by SDS-PAGE analysis (Fig 2 and S1 Fig). We found that 0.1 mg of Endo-CC1 and 0.045 mg of Endo-CC2 were purified from 250 ml of each *E*. *coli* culture.

**Fig 3:**
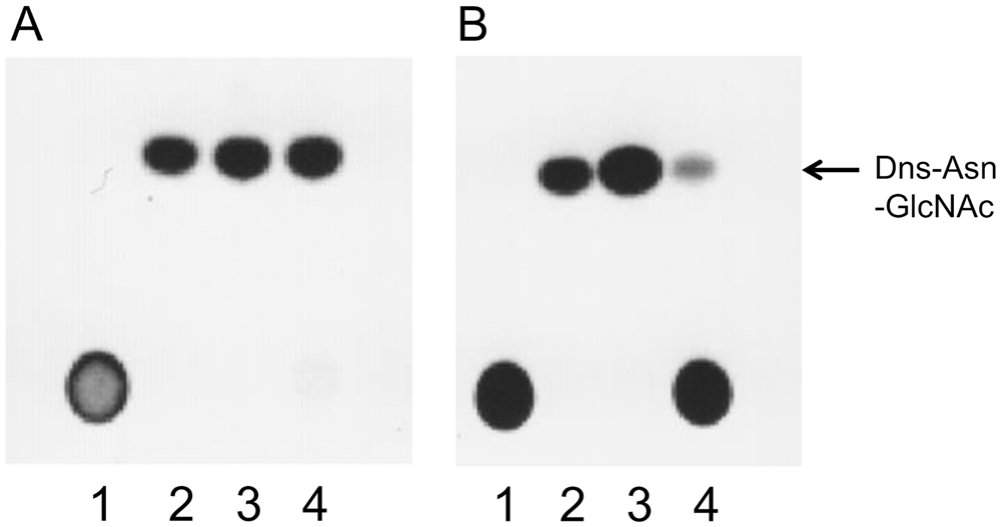
Analyses of hydrolase activity of Endo-CCs by TLC. Reaction mixtures containing Dns-Man_5_GlcNAc_2_Asn or Dns-sialylglyco-Asn and Endo-CC1 (A) or Endo-CC2 (B) were analyzed by TLC. Lane 1, Dns-sialylglyco-Asn without any added enzyme; lane 2, Dns-Asn-GlcNAc without any added enzyme; lane 3, Dns-Man_5_GlcNAc_2_Asn with added enzyme; lane 4, Dns-sialylglyco-Asn with added enzyme.

Next, to investigate whether these purified enzymes would show ENGase activity, we used Man_5_GlcNAc_2_Asn-Dns and biantennary sialylglyco-Asn-Dns (Dns-SG) as model high-mannose and complex type oligosaccharides, respectively. Although both Endo-CC1 and Endo-CC2 were able to hydrolyze these two substrates, the hydrolytic activity of Endo-CC2 for the complex type oligosaccharide was clearly less than that of Endo-CC1 (Fig 3).

To further analyze the substrate specificities of Endo-CC1 and Endo-CC2, we next measured the relative activity of each enzyme using several types of PA-oligosaccharides as substrates, and the hydrolysis products were analyzed by HPLC. The results are summarized in Table 1, in which the relative activity of each enzyme for M8 was set as 100 because Endo-M exhibits highest activity for this substrate [15]. Both Endo-CC1 and Endo-CC2 exhibited the hydrolase activity for high-mannose type and biantennary complex type oligosaccharides like Endo-M. Although Endo-M hydrolyzed asialobiantennary oligosaccharide containing bisecting GlcNAc, neither Endo-CC1 nor Endo-CC2 did. None of these enzymes could hydrolyze asialotriantennary nor asialotetraantennary oligosaccharides. Overall, both Endo-CC1 and Endo-CC2 exhibited relatively similar substrate specificity as the Endo-M, although relative activities of Endo-CC1 and Endo-CC2 for high-mannose type oligosaccharides were comparatively higher than those of Endo-M. Collectively, considering that the hydrolytic activity of Endo-CC1 on Dns-SG was higher than that of Endo-CC2 (Fig 3), we postulated that Endo-CC1 would be a potential candidate with properties similar to that of Endo-M.

**Table 1:** Relative hydrolase activity of Endo-CCs and Endo-M.

PA-oligosaccharide	Relative activity (%)^a^		
	Endo-CC1	Endo-CC2	Endo-M^c^
PA-trimannosyl core	119	179	19.5
PA-oligomannoside (M5)	84.1	55.9	15.4
PA-oligomannoside (M6)	153	181	74.0
PA-oligomannoside (M8)	100	100	100
PA-oligomannoside (M9)	42.4	109	66.5
PA-agalactobiantennary	20.8	8.2	4.4
PA-asialobiantennary	17.9	12.5	13.3
PA-sialobiantennary	11.2	6.0	7.0
PA-asialobiantennary (bisecting GlcNAc)	ND^b^	ND^b^	2.0
PA-asialotriantennary	ND^b^	ND^b^	ND^b^
PA-asialotetraantennary	ND^b^	ND^b^	ND^b^

Various PA-oligosaccharides were used as a substrate for these assays.

^a^ The relative activity value of each enzyme for a given PA-oligosaccharide was calculated with respect to its value for M8, which was set at 100.

^b^ ND, not detectable.

^c^ Data are taken from [15].

Then, we further characterized its enzymatic properties using Dns-SG as a substrate. We found that the optimum pH of Endo-CC1 is 7.5 (S2A Fig), which is higher than the optimum pHs of other ENGases. Next, we determined the Km of Endo-CC1 for Dns-SG and found it to be 1.9 mM (S2B Fig). Since the Km of Endo-M for Dns-asialo-transferrin glycopeptide was reported to be 2.0 mM, our result suggested that the hydrolytic activity for biantennary complex type oligosaccharide of Endo-CC1 might be similar to that of Endo-M [12]. We also checked the thermostability of Endo-CC1 and found that its activity remained unchanged even after incubation at 50°C for 10 min (S2C Fig).

### Hydrolytic activity of Endo-CC1 on glycoproteins

**Fig 4:**
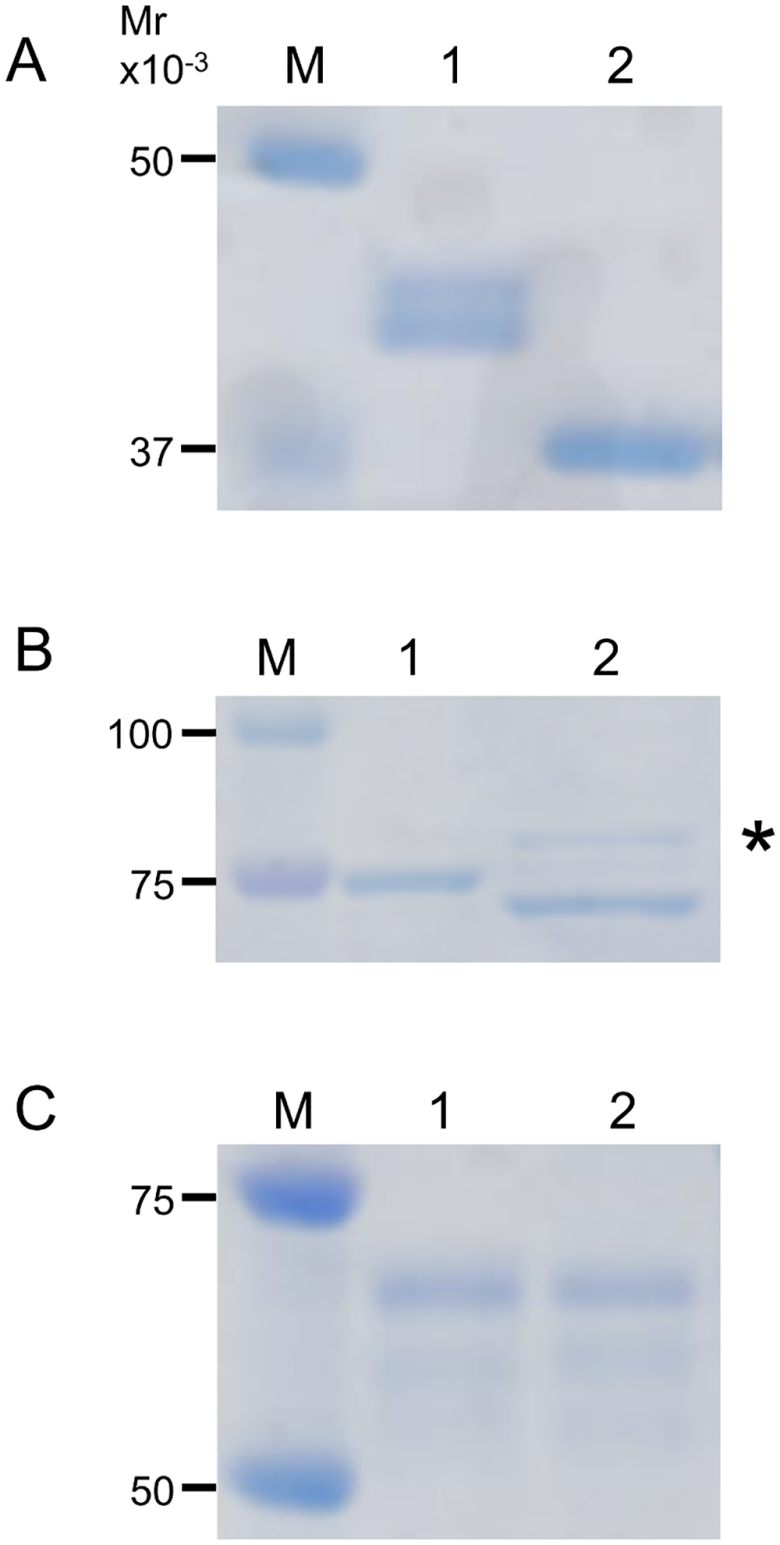
Hydrolase activity of Endo-CC1: effect on glycoproteins. SDS-PAGE analysis of reaction mixtures containing 1 μg of RNase B (A), human transferrin (B) and fetuin (C) treated with or without Endo-CC1. The double bands seen in the lane 1 of (A) are likely due to different modifications of *N*-glycosylation on RNaseB. The protein band marked with an asterisk in (B) represents Endo-CC1. Lane M, molecular weight markers; lane 1, negative control (without Endo-CC1); lane 2, reaction product (with Endo-CC1).

We further tested whether Endo-CC1 could hydrolyze the *N*-linked glycans of glycoproteins. RNase B, human transferrin and fetuin were chosen as glycoproteins containing high-mannose, sialobiantennary and sialotriantennary type oligosaccharides, respectively. We found that Endo-CC1 was able to hydrolyze RNase B (Fig 4A) and human transferrin (Fig 4B), but not fetuin (Fig 4C), suggesting that Endo-CC1 can hydrolytically remove both high-mannose and biantennary complex type oligosaccharides on glycoproteins.

### Transglycosylation activity of Endo-CC1 mutants

Since Endo-CC1 can hydrolyze the biantennary complex type oligosaccharide, we assumed that Endo-CC1 can transfer the biantennary complex type oligosaccharide onto the GlcNAc residue of glycoproteins. It was previously reported that Asn-175 of Endo-M is an important amino acid residue required for the catalysis [19]. As the corresponding amino acid residue in Endo-CC1 is Asn-180 (N180), we replaced the N180 residue of Endo-CC1 individually with 19 other amino acid residues by PCR mutagenesis and purified the respective mutant proteins from *E*. *coli* following the same protocol that was used for the purification of wild-type Endo-CC1 (see above). Hydrolytic activity of all 20 recombinant proteins was analyzed by HPLC using Dns-SG as the substrate. Results shown in Table 2 showed that N180 is indeed the critical residue that is responsible for the hydrolytic activity of Endo-CC1.

**Table 2:** Relative hydrolase activity of recombinant wild-type and mutant Endo-CC1s.

Endo-CC1	Relative activity (%)^a^
WT (N180)	100
A	0.5
C	2.1
D	5.8
E	3.3
F	0.1
G	0.4
H	8.7
I	4.0
K	0.2
L	0.6
M	12.0
P	0.8
Q	16.9
R	ND^b^
S	1.0
T	1.0
V	3.4
W	ND^b^
Y	ND^b^

The Asn 180 (N180) residue of the wild-type (WT) Endo-CC1 was replaced individually with the one of the remaining 19 amino acid residues (indicated above using single-letter code for each amino acid residue) and the relative hydrolase activity of each mutant was determined using purified recombinant mutant and Dns-DG as the substrate.

^a^ The relative activity of the WT Endo-CC1 was set as 100.

^b^ ND, not detectable.

**Fig 5:**
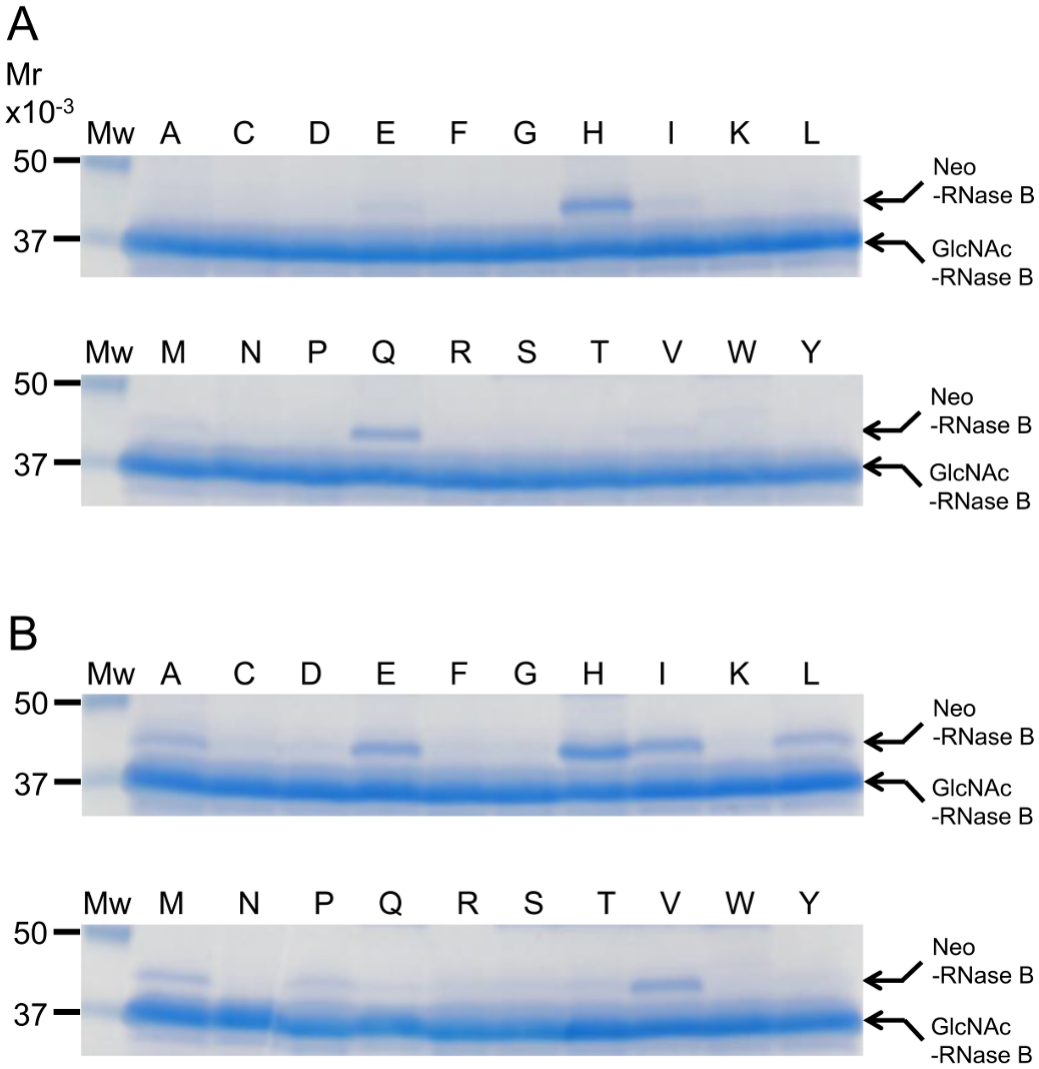
Transglycosylation activity of Endo-CC1^N180X^. SDS-PAGE analysis of transglycosylation reaction mixtures that contained deglycosylated RNase B, indicated purified Endo-CC1^N180X^ mutant (where X is the altered amino acid residue in the point mutant) and complex type oligosaccharide derived from SGP: 1 h incubation (A) and 12 h incubation (B). The single letter label on each lane of the gel indicates the point mutant that was used in the transglycosylation assay; thus, lane labeled N is the wild-type Endo-CC1. Mw, molecular weight markers.

Next, to determine whether Endo-CC1 has the transglycosylation activity, we performed a transglycosylation assay using deglycosylated RNase B (GlcNAc-RNase B), which was prepared by hydrolyzing RNase B with Endo-CC1, and biantennary complex type oligosaccharide derived from SGP, as the acceptor and donor substrates, respectively. Under the reaction condition used in this study, the wild-type Endo-CC1 could not transfer the complex type sialobiantennary oligosaccharide onto the GlcNAc-RNase B (Fig 5A, lane marked as N). In contrast, both N180H and N180Q mutants appeared to have transferred the oligosaccharide onto the GlcNAc-RNase B, as a new protein band appeared on the SDS-PAGE gels of respective reaction mixtures incubated for 1 h (Fig 5A, lanes marked as H and Q); we named this new protein as Neo-RNase B. After 12 h incubation of the reaction mixtures, the N180Q mutant cleaved the oligosaccharide off the Neo-RNase B, whereas the N180H mutant did not cleave the oligosaccharide off the Neo-RNase B (Fig 5B).

**Fig 6:**
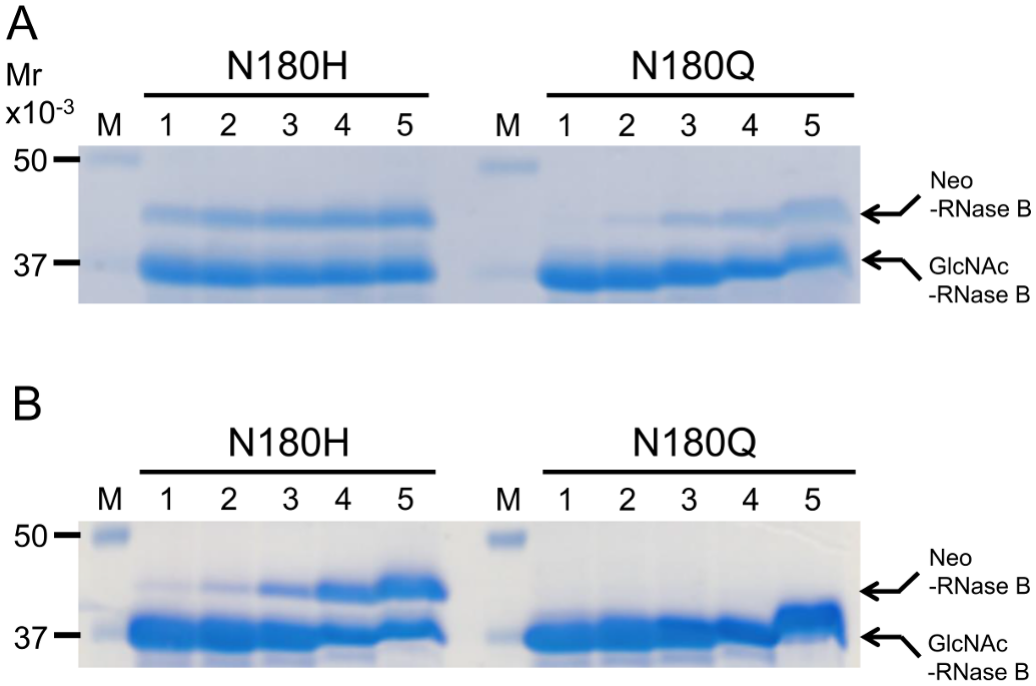
Transglycosylation of deglycosylated RNase B by Endo-CC1^N180H^ and End-CC1^N180Q^ at various SGP concentration. SDS-PAGE analysis of transglycosylation reaction mixtures that contained deglycosylated RNase B, purified Endo-CC1 mutant (Endo-CC1^N180H^ or EndoCC1^N180Q^) and different amounts of complex type oligosaccharide derived from SGP: 1 h incubation (A) and 12 h incubation (B). Amount of SGP used in the reaction mixture (μg): Lane 1, 62.5; lane 2, 125; lane 3, 250; lane 4, 500; lane 5, 1000. Note that in lanes 5, mobility shifts were seen likely due to a large amount of SGP included in the samples.

To determine the effect of donor substrate concentration on the synthesis of Neo-RNase B, we repeated the transglycosylation experiment using various amounts of SGP. As shown in Fig 6A, after 1 h incubation both N180H and N180Q mutants produced more Neo-RNase B as the amount of SGP used in the reaction mixture was increased. After 12 h incubation, the amount of Neo-RNase B produced in the N180H containing reaction mixture was found to have increased as the amount of SGP increased; in contrast, no Neo-RNase B was detected in the N180Q mutant containing reaction mixture after 12 h incubation (Fig 6B).

**Fig 7:**
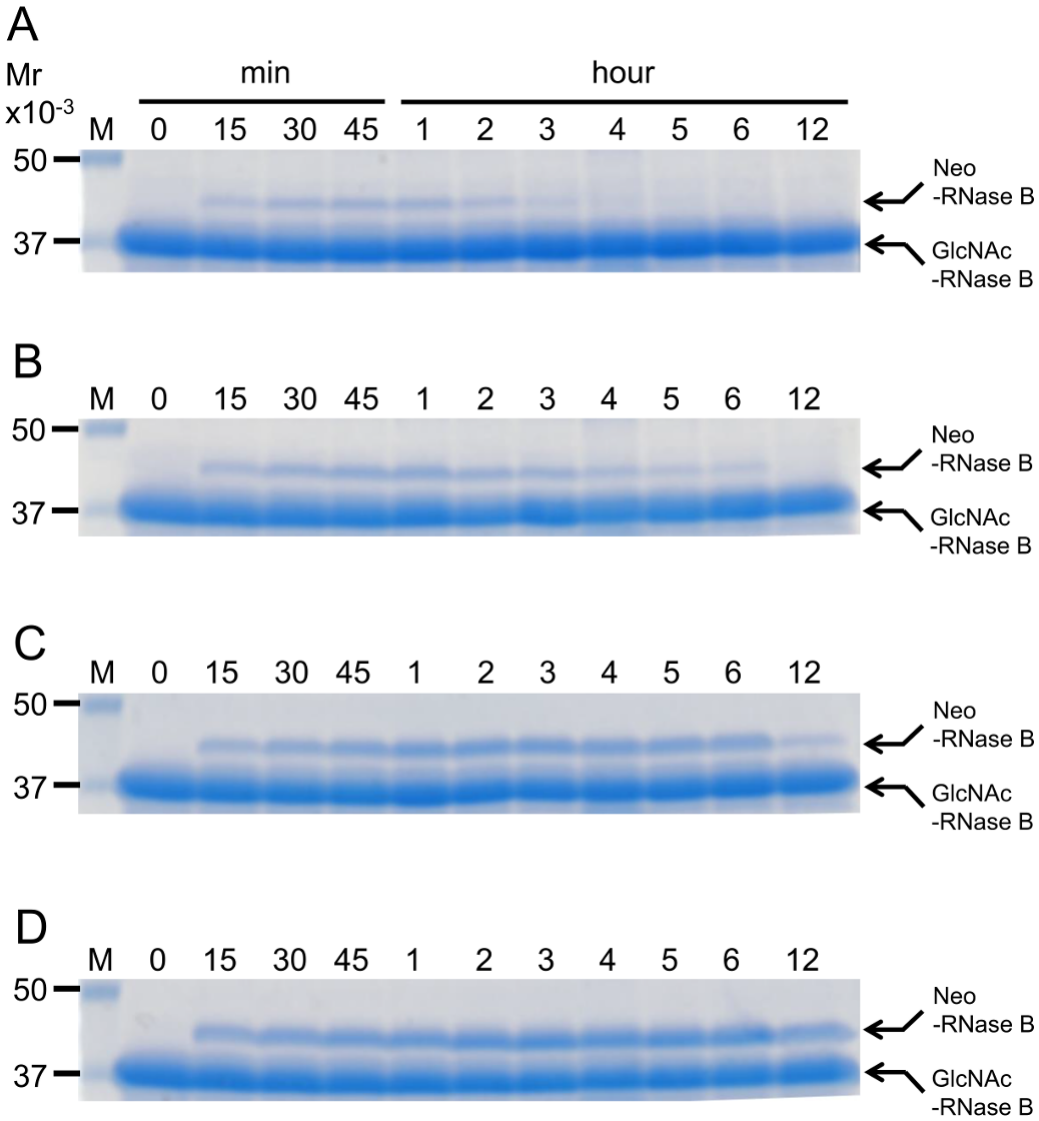
Time course of transglycosylation by Endo-CC1^N180Q^ and Endo-CC1^N180H^. Time course of transglycosylation of deglycosyalted RNase B by either End-CC1^N180Q^ or Endo-CC1^N180H^ was examined using different amount of complex type oligosaccharide derived from SGP. (A) Endo-CC1^N180Q^ and 62.5 μg SGP. (B) Endo-CC1^N180Q^ and 250 μg SGP. (C) Endo-CC1^N180H^ and 62.5 μg SGP. (D) Endo-CC1^N180H^ and 250 μg SGP.

Lastly, we analyzed the transglycosylation of the GlcNAc-RNase B by N180Q and N180H mutants as a function of time. The N180Q mutant first generated Neo-RNase B, which gradually disappeared at later time points, probably because of the remaining hydrolase activity of the N180Q mutant (Fig 7A and 7B). On the other hand, the N180H mutant kept on producing more Neo-RNase B as the time progressed and the generated Neo-RNase B was relatively stable during the time course (Fig 7C and 7D). Taken together, we conclude that the N180H mutant of Endo-CC1 has the desired transglycosylation property.

### Analysis of *N*-glyans of Neo-RNase B

**Fig 8:**
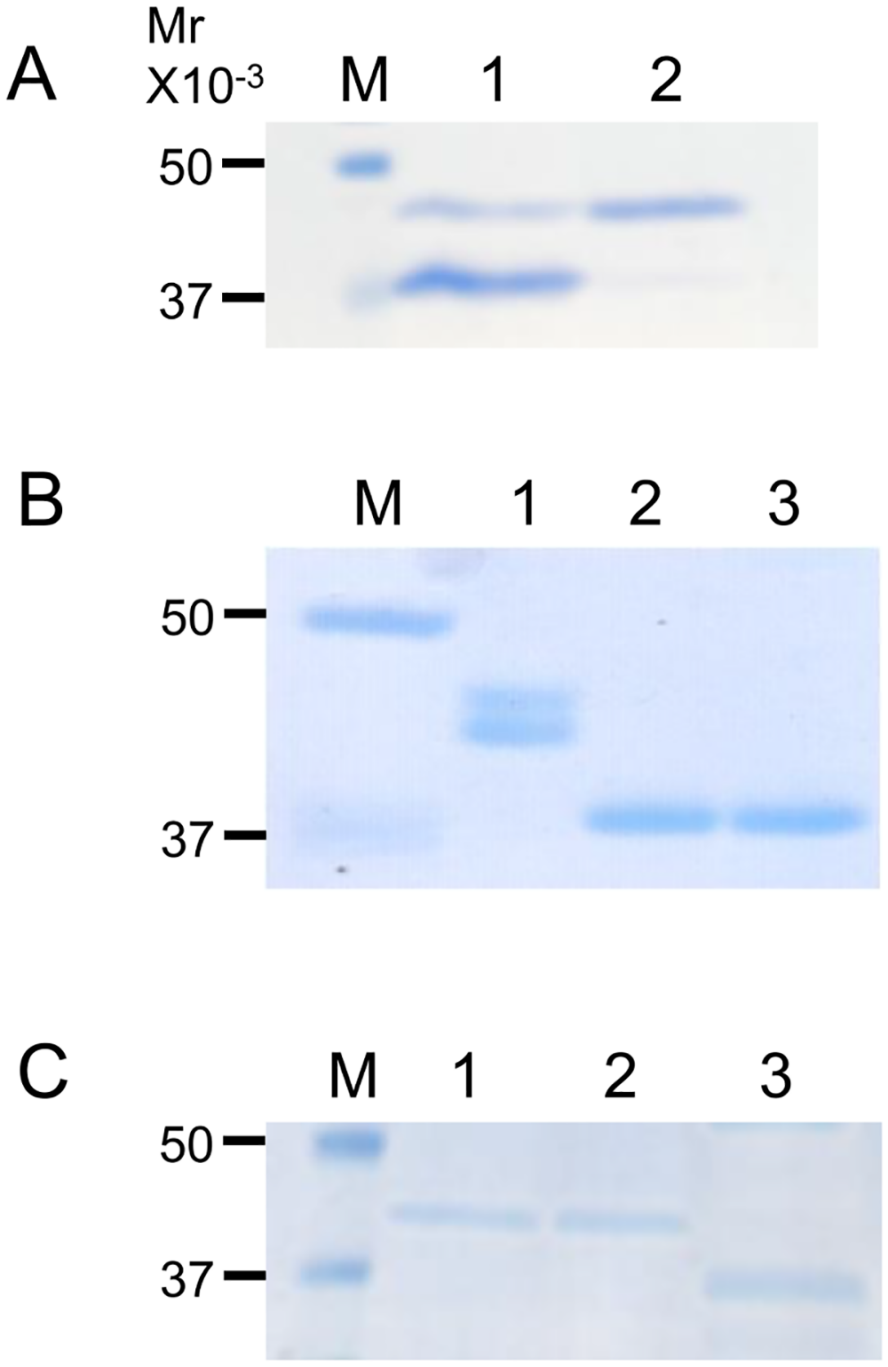
SDS-PAGE analyses of purified Neo-RNase B. (A) Neo-RNase B was purified with a Con A column. Lane M, molecular weight markers; lane 1, before purification; lane 2, after purification. (B) Lane M, molecular weight markers; lane 1, native RNase B (control); lane 2, native RNase B treated with Endo-A; lane 3, native RNase B treated with Endo-CC1. Note that the double bands seen in the lane 1 are likely due to different modifications of *N*-glycosylation on RNaseB. (C) Lane M, molecular weight markers; lane 1, purified Neo-RNase B (control); lane 2, purified Neo-RNase B treated with Endo-A; lane 3, purified Neo-RNase B treated with Endo-CC1.

To confirm whether the formed Neo-RNase B indeed had the biantennary complex oligosaccharide attached to it, we first purified the Neo-RNase B from the transglycosylation mixture, which also contained GlcNAc-RNase B, using a Con A column (Fig 8A). Since Endo-A can act on high-mannose type oligosaccharide, but not on biantennary complex type oligosaccharide, we next treated native RNase B, a glycoprotein that is modified with high-mannose type oligosaccharide, with Endo-A and Endo-CC1, and found that both of them cleaved native RNase B (Fig 8B). In contrast, Endo-CC1, but not Endo-A, was able to cleave the purified Neo-RNase B (Fig 8C), suggesting that the purified Neo-RNase B indeed contains biantennary complex type oligosaccharide, which was transferred onto the GlcNAc-RNase B by Endo-CC1^N180H^.

## Discussion

In this study, we identified two novel ENGases, Endo-CC1 and Endo-CC2, from the basidiomycete fungus *C*. *cinerea*. Both enzymes can catalyze the hydrolysis of high-mannose and biantennary complex type oligosaccharides, and the substrate specificity of Endo-CC1 and Endo-CC2 resembles that of Endo-M, although there were certain differences in relative activities for high-mannose oligosaccharides and asialobiantennary oligosaccharide containing bisecting GlcNAc. Mutational analysis of Endo-CC1, which is expressed more easily in *E*. *coli* than Endo-CC2, further demonstrated that the Endo-CC1^N180H^ mutant can effectively transfer the sialobiantennary complex type oligosaccharide onto the GlcNAc-RNase B to produce a glycosylated form of RNase B, termed here as Neo-RNase B.

Endo-M is one of the most used enzymes for assessing oligosaccharides on pharmaceutical glycoproteins. However, commercially available Endo-M is relatively expensive, and thus warranting the need for another enzyme that would not only possess Endo-M-like enzymatic properties, but could also be purified easily in large amounts. We found that Endo-CC1 is comparatively available more readily than Endo-M, as Endo-CC1, but not Endo-M, could be prepared easily from *E*. *coli* grown at 30°C [15]. In addition, Endo-CC1 is thermostable at 50°C for 10 min and may have a similar affinity for the biantennary complex type oligosaccharides to Endo-M. The optimum working pH of Endo-CC1 is 7.5, which is different from that of other ENGases (normally between 5–6). Taken together, we reason that Endo-CC1 could be a less expensive substitute for evaluating oligosaccharides on pharmaceutical glycoproteins, for which Endo-M is the currently used enzyme.

In order to create an Endo-CC1 mutant that has higher activity for transferring biantennary complex type oligosaccharide, we performed point mutation analysis of the catalytically important amino acid residue Asn-180 of Endo-CC1. In Endo-A and Endo-M, mutagenizing the catalytic residue from Asn to Ala caused significant reduction in their hydrolytic activity [19,20]. In addition, the N175Q mutant of Endo-M was shown to exhibit better transglycosylation activity compared to the wild-type enzyme [21]. This is probably because Q contains an additional methylene residue that might help this mutant not only to sustain the hydrolase activity but also to acquire more transglycosylation activity. Among all the Asn-180 point mutants of Endo-CC1, we found that Endo-CC1^N180H^ exhibits the most desired transglycosylation activity (i.e., the ability to efficiently transfer and retain sialobiantennary complex type oligosaccharide onto deglycosylated RNase B).

Recently, sugar oxazolines that can mimic the transition state of the reaction have been used for transglycosylation [7]. By using highly active sugar oxazolines, it was reported that both Endo-A and Endo-M can efficiently transfer complex type *N*-glycans [19–23]. In this study, we used SGP as a donor substrate and found that the Endo-CC1^N180H^ mutant is best able to transfer sialobiantennary complex oligosaccharide onto deglycosylated RNase B. Since the hydrolase activity is not required if sugar oxazoline is used as a donor substrate, it might be possible that other Endo-CC1 mutants, apart from the Endo-CC1^N180H^, would exhibit higher transglycosylation activity. Therefore, we are currently investigating the transglycosylation activity of all Endo-CC1 mutants using sugar oxazolines.

In summary, we found two novel ENGases, one of which, Endo-CC1, can be easily prepared as a recombinant protein, and it can hydrolyze and transfer biantennary complex type oligosaccharide. These features are highly desired for analyzing the oligosaccharide contents of pharmaceutical glycoproteins and also for remodeling of heterogeneous oligosaccharides on glycoproteins into homogenous oligosaccharide, which is crucial for their consistent bioactivity.

## Supporting Information

S1 Fig Images of SDS-PAGE gels to confirm purification of Endo-CCs. (TIF)

S2 Fig Enzymatic properties of Endo-CC1. (TIF)

S3 Fig Images of original SDS-PAGE gels cropped and used in main figures. (TIF)

S1 Table Primers used in this study. (DOCX)
