# Supplementary figures and images for: Transglycosylation Activity of Glycosynthase Mutants of Endo-β-N-Acetylglucosaminidase from Coprinopsis cinerea
Source: PLoS One. 2015 Jul 21;10(7):e0132859. doi: 10.1371/journal.pone.0132859 (PMC4510386; doi:10.1371/journal.pone.0132859)

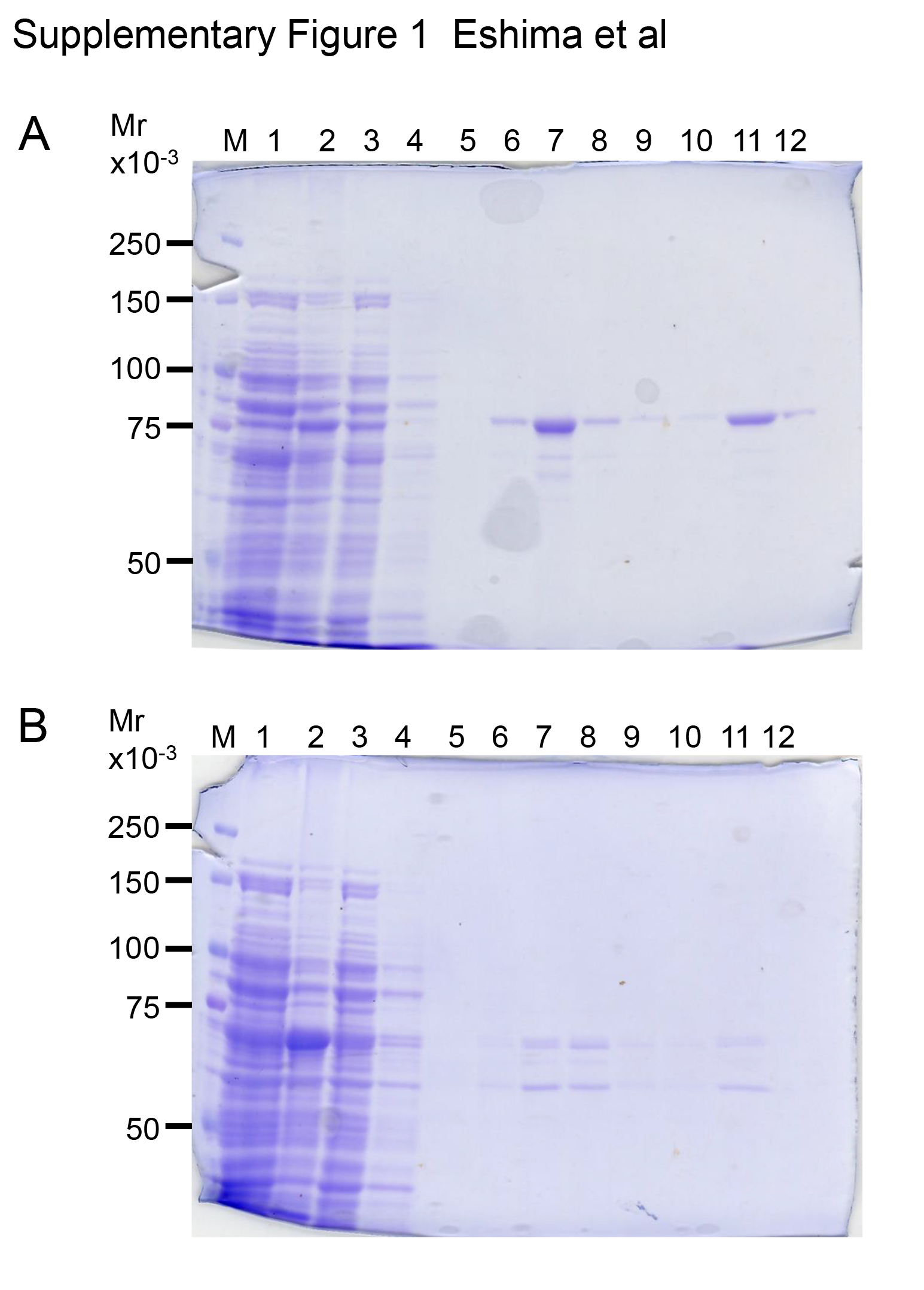

Supplement: S1 Fig — (TIF) [file pone.0132859.s001.tif]

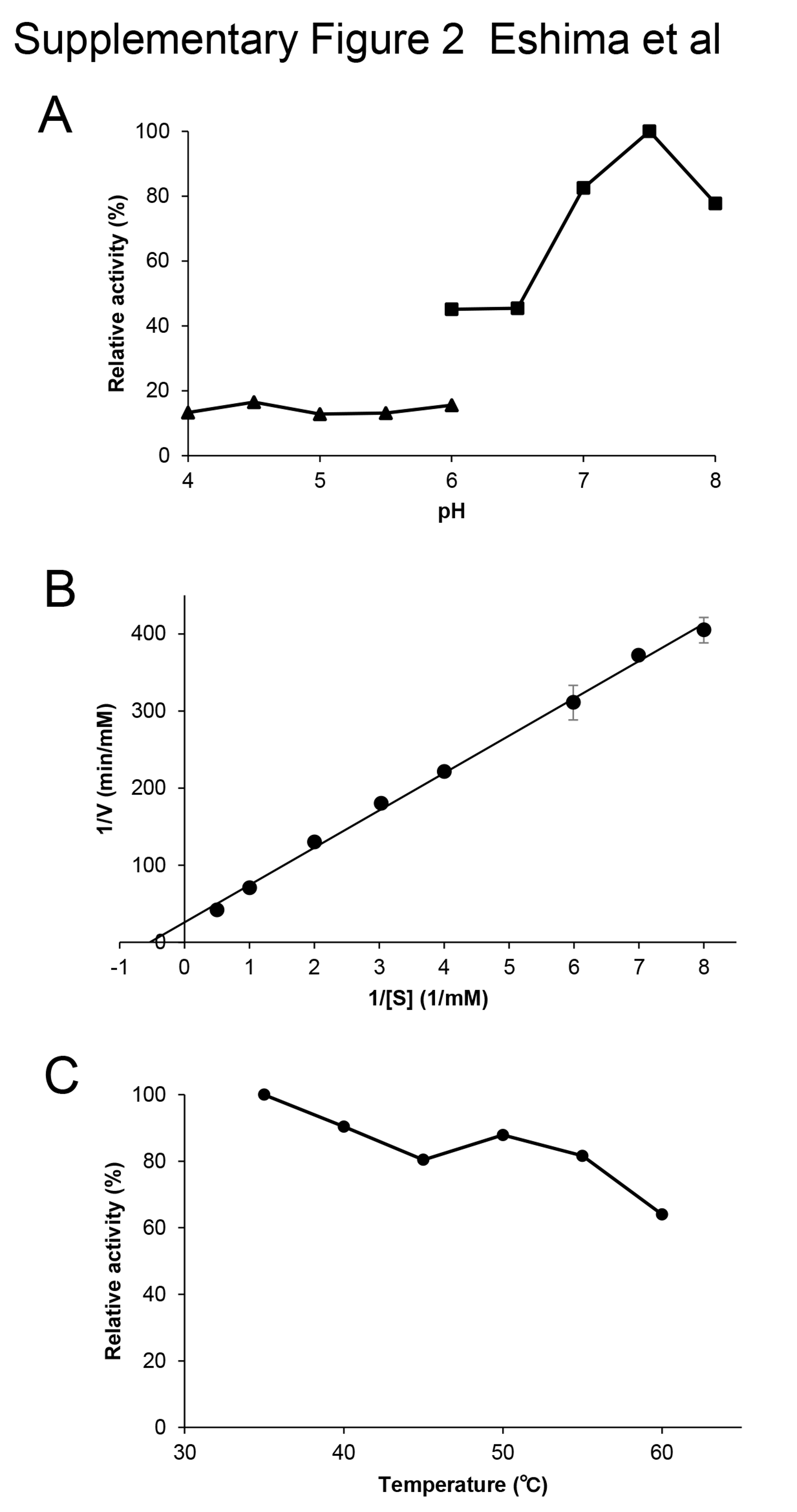

Supplement: S2 Fig — (TIF) [file pone.0132859.s002.tif]

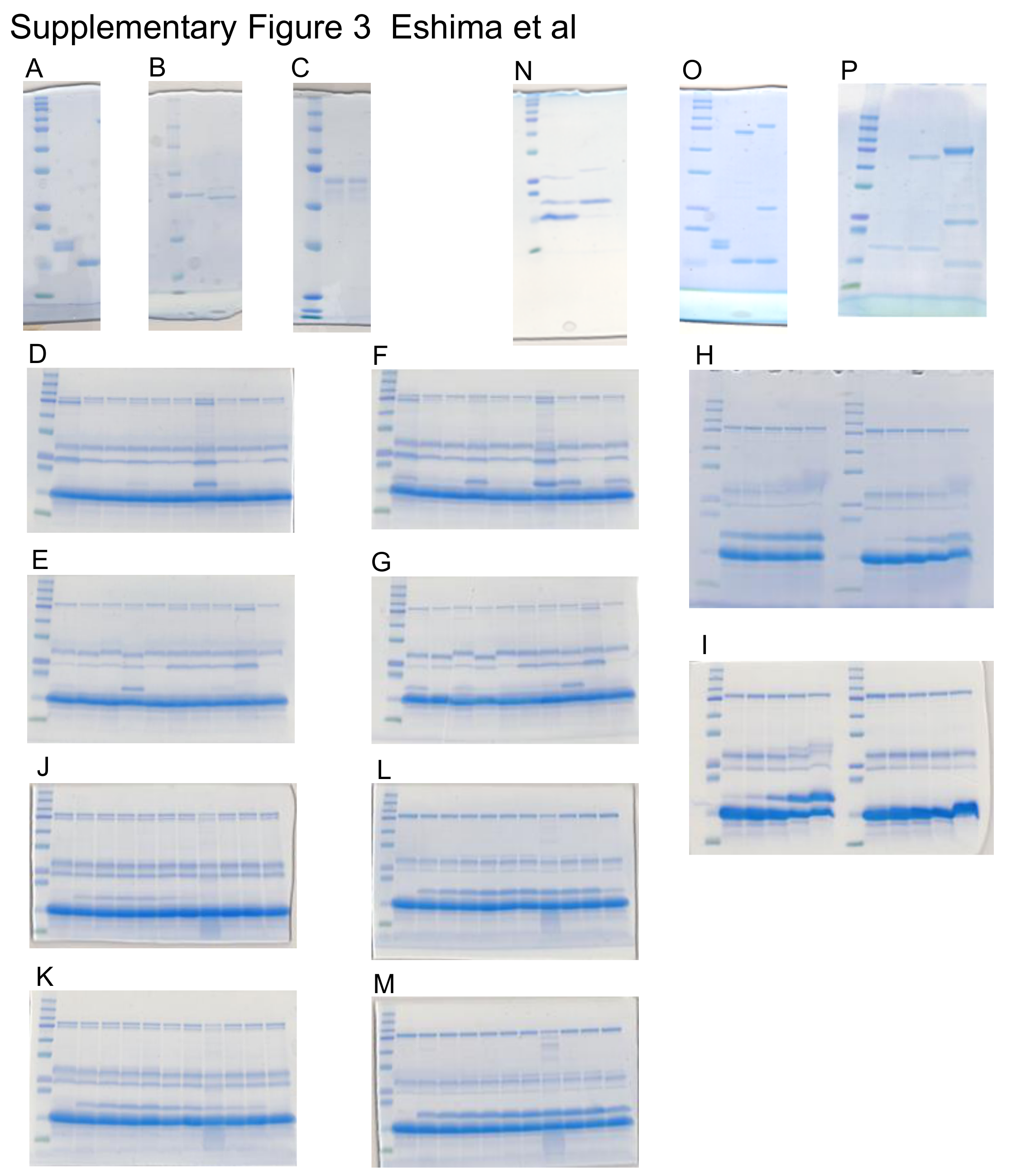

Supplement: S3 Fig — (TIF) [file pone.0132859.s003.tif]
