# Supplementary material for: Transglycosylation Activity of Glycosynthase Mutants of Endo-β-N-Acetylglucosaminidase from Coprinopsis cinerea
Source: PLoS One. 2015 Jul 21;10(7):e0132859. doi: 10.1371/journal.pone.0132859 (PMC4510386; doi:10.1371/journal.pone.0132859)
Supplement: S1 Table — (DOCX) [file pone.0132859.s004.docx]

**Supplementary Table 1. Primers used in this study.**

| Target | Sequence (5’ to 3’; upper, forward; lower, reverse) |
| --- | --- |
| pET-23b  Endo-CC1 cDNA  Endo-CC1^N180A^ cDNA  Endo-CC1^N180C^ cDNA  Endo-CC1^N180D^ cDNA  Endo-CC1^N180E^ cDNA  Endo-CC1^N180F^ cDNA  Endo-CC1^N180G^ cDNA  Endo-CC1^N180H^ cDNA  Endo-CC1^N180I^ cDNA  Endo-CC1^N180K^ cDNA  Endo-CC1^N180L^ cDNA  Endo-CC1^N180M^ cDNA  Endo-CC1^N180P^ cDNA  Endo-CC1^N180Q^ cDNA  Endo-CC1^N180R^ cDNA  Endo-CC1^N180S^ cDNA  Endo-CC1^N180T^ cDNA  Endo-CC1^N180V^ cDNA  Endo-CC1^N180W^ cDNA  Endo-CC1^N180Y^ cDNA  Endo-CC2 cDNA | CTCGAGCACCACCACCACCACCACTGAGAT  CATATGTATATCTCCTTCTTAAAGTTAAAC  GGAGATATACATATGCCTATCGCTGGGAAGAAGTTCCAC  GTGGTGGTGCTCGAGCTTGGCGACGGAGATATCGCCCTT  ctcaaccgcgagcagccaaccatcgaa  ctgctcgcggttgagattggtctgcag  ctcaacgcagagcagccaaccatcgaa  ctgctcaacgttgagattggtctgcag  ctcaacatcgagcagccaaccatcgaa  ctgctcgatgttgagattggtctgcag  ctcaacttcgagcagccaaccatcgaa  ctgctcgaagttgagattggtctgcag  ctcaacaaagagcagccaaccatcgaa  ctgctctttgttgagattggtctgcag  ctcaacgccgagcagccaaccatcgaa  ctgctcggcgttgagattggtctgcag  ctcaacatggagcagccaaccatcgaa  ctgctccatgttgagattggtctgcag  ctcaacaatgagcagccaaccatcgaa  ctgctcattgttgagattggtctgcag  ctcaactttgagcagccaaccatcgaa  ctgctcaaagttgagattggtctgcag  ctcaaccaggagcagccaaccatcgaa  ctgctcctggttgagattggtctgcag  ctcaaccatgagcagccaaccatcgaa  ctgctcatggttgagattggtctgcag  ctcaaccgggagcagccaaccatcgaa  ctgctcccggttgagattggtctgcag  ctcaacctggagcagccaaccatcgaa  ctgctccaggttgagattggtctgcag  ctcaacgcggagcagccaaccatcgaa  ctgctccgcgttgagattggtctgcag  ctcaacgctgagcagccaaccatcgaa  ctgctcagcgttgagattggtctgcag  ctcaacggtgagcagccaaccatcgaa  ctgctcaccgttgagattggtctgcag  ctcaaccacgagcagccaaccatcgaa  ctgctcgtggttgagattggtctgcag  ctcaacccagagcagccaaccatcgaa  ctgctctgggttgagattggtctgcag  ctcaacatagagcagccaaccatcgaa  ctgctctatgttgagattggtctgcag  GGAGATATACATATGCCAGTCCGCGGAACGGTACCCCAA  GTGGTGGTGCTCGAGTCCATCCGTTCTTGGTTTGGGGAA |
